# Simulation and Experimental Substantiation of the Thermal Properties of Non-Autoclaved Aerated Concrete with Recycled Concrete Powder

**DOI:** 10.3390/ma15238341

**Published:** 2022-11-23

**Authors:** Xiaosong Ma, Hao Li, Dezhi Wang, Chunbao Li, Yongqi Wei

**Affiliations:** 1 School of Civil and Water Conservancy Engineering, Ningxia University, Yinchuan 750021, China; 2 Department of Civil Engineering, China University of Petroleum (East China), Qingdao 266580, China; 3 Key Laboratory of Advanced Civil Engineering Materials of Ministry of Education, Tongji University, Shanghai 201804, China; 4 School of Materials Science and Engineering, Tongji University, Shanghai 201804, China

**Keywords:** non-autoclaved aerated concrete, thermal conductivity, COMSOL simulation, pore size distribution, image-based analysis

## Abstract

Non-autoclaved aerated concrete (NAAC) is a two-phase material with a concrete matrix and air, exhibits good thermal insulation performance and shows good potential in the insulating construction industry. In this study, recycled concrete fine powder was used as an auxiliary cementing material, and the NAAC with different porosity and distribution was fabricated by the non-autoclaved method at different curing temperatures. The effect of porosity on the thermal conductivity and mechanical strength of NAAC is analyzed by experimental tests. A prediction method of thermal conductivity combining pore structure reconstruction and numerical simulation was proposed, which is established by two steps. Firstly, the pore size distributions of NAAC with different porosities were characterized by stereology image analyses. Secondly, the thermal conductivity prediction model based on the pore structure information was established by a COMSOL steady-state heat transfer module. The thermal conductivity results of COMSOL simulations were compared with the experiments and other theoretical models to verify the reliability of the model. The model was used to evaluate the effect of porosity, pore size distribution and the concrete matrix’s thermal conductivity on the thermal conductivity of NAAC; these are hard to measure when only using laboratory experiments. The results show that with the increase in curing temperature, the porosity of NAAC increases, and the number and volume proportion of macropores increase. The numerical results suggest that the error between the COMSOL simulations and the experiments was less than 10% under different porosities, which is smaller than other models and has strong reliability. The prediction accuracy of this model increases with the increase in NAAC porosity. The steady thermal conductivity of NAAC is less sensitive to the distribution and dispersion of pore size in a given porosity. With the increase in porosity, the thermal conductivity of NAAC is linearly negatively correlated with that of the concrete matrix, and the correlation is close to 1.

## 1. Introduction

With the burgeoning call towards environmental-friendliness and sustainable development strategies, it is necessary to explore effective solutions for energy efficiency and carbon emission reduction in the construction. Heating and cooling costs of a building are the main parts of operating costs during the life cycle of the building and also one of the main sources of carbon emissions [1]. Hence, excellent insulation building materials are considered important in the building’s design and construction.

Aerated concrete (AC) is a lightweight porous material made by introducing expansion agents into a slurry mixed with binders, supplementary cementitious material, fine aggregate, admixture and water [2,3,4]. Due to its small bulk density, good thermal insulation and fire resistance, AC attracted extensive interest in the field of energy-efficient and thermal insulation [5,6,7]. According to the production method, the AC can be divided into autoclaved aerated concrete (AAC) and non-autoclaved aerated concrete (NAAC). The production of NAAC does not require an environment of saturated steam under pressure, which is expensive and requires high energy consumption [8,9,10]. Simultaneously, a large amount of recycled material can be reused as raw materials for AC production, revealing the great potential in increasing the utilization rate of industrial waste [11,12,13,14]. As one of the widely used building materials in modern construction, the production of concrete has generated a large amount of waste concrete due to remodeling and demolition processes [15,16]. Waste concrete is reused in low-value operations or end up in landfills, which can cause a serious waste of resources. Furthermore, the current production of cement clinkers is accompanied by a large amount of energy consumption and carbon dioxide emissions. Many researchers proved that recycled concrete fines can partially replace traditional cementitious materials in concrete production [17,18,19,20]. Consequently, incorporating recycled concrete aggregates in NAAC production is considered to be a cost-effective means for solving the construction waste disposal challenges and achieving environmental sustainability.

Many researchers studied the characteristics of concrete produced with recycled concrete aggregates, including mechanical strength, crack resistance, porosity and water absorption [21,22]. Nevertheless, the research on the thermal conductivity of NAAC mixed with recycled concrete fine powder is very limited. In the past few decades, most studies have focused on examining the influence of the mix ratio on the thermal conductivity of porous concrete in order to obtain improved thermal insulation properties [23,24,25,26,27]. For example, Qu et al. [23] showed that the thermal conductivity of AAC is mainly influenced by its dry density. Narayanan [24] and Jerman et al. [25] reported that the moisture content is a key factor affecting the thermal conductivity of AC, and the higher value of moisture contents led to higher thermal conductivities. Jeong et al. [26] investigated the effect of water/cement ratios on the thermal conductivity of porous concrete with coal bottom ash. As a result of the research study, the authors found that thermal conductivities were significantly reduced by increases in the water/cement ratio.

The NAAC is a two-phase material with a concrete matrix and air, and one of its most important features is its internal porous structure. The macropores are formed from the chemical reaction between expansion agent and cementitious mixtures. The gas overflow after the addition of the expansion agent forms millions of uniformly sized and evenly distributed air voids in the concrete matrix, which are much bigger than the ordinary concrete [2,28,29]. In recent years, a large number of studies proved that the thermal insulating ability of porous concrete is significantly affected by its pore structure [30,31]. To intensively study the heat transfer performance of porous materials, many studies attempted to link thermal conductivity with its pore structure parameters [32,33]. The constitutive models of two-phase materials between the pore structure and thermal behaviors either have been established by many researchers in an analytical or empirical way to estimate the effective thermal conductivity [34,35,36]. For example, the parallel–series model and Campbell-Allen model are commonly used thermal conductivity models. Li et al. [35] established a predicting model for a simple two-phase inclusion-matrix system based on an effective medium and mean-field theories and focused on the effect of pore shape on thermal conductivity. Othuman et al. [36] proposed an analytical model of thermal conductivity with respect to foam concrete based on the assumed internal structure (porous) and constituents (cement, water and air). These models can quantitatively establish the relationship between porosity and thermal conductivity by using the thermal conductivity of two phases (concrete matrix and air) in porous concrete and the corresponding volume fraction. However, due to the fact that the microstructure of porous concrete is complex and the hypotheses of two-phase homogeneous material is oversimplified, the real properties of porous concrete are not been expressed exactly. Generally, all of the above models simply focused on changes in thermal conductivities caused by pore volume differences but neglected the effect of parameters such as pore size and distribution.

In fact, it is usually difficult to take the geometric details of the pores into consideration in experimental studies. Based on the reasonable calculation model and enough accurate data of the NAAC’s internal features, numerical simulations can quantitatively establish the relationship between pore structure and thermal conductivity, and these simulations have become one of the important methods for the prediction of effective thermal conductivity. For example, Miled et al. [37] used five homogenization models to predict foam concrete’s thermal conductivity, and the experimental data and FEM simulations are used to identify the best model. Qin et al. [38] used the steady-state method to numerically simulate the thermal conductivity of the foam glass. Ding et al. [39] used COMSOL simulations to study the relationship between thermal conductivity and the porosity of concrete materials. For most existing numerical models, the pore structure in NAAC is reproduced by random methods; thus, the influence of production process and material properties differences on the real pore structure of NAAC is usually ignored. However, this issue is of great importance for creating appropriate pore structure for NAAC to predict thermal conductivities; thus, the prediction results of the numerical model based on the random pore generation method usually lack stability. A large number of pores in NAAC are macro-pores, and common pore structure characterization methods such as scanning electron microscope (SEM), mercury injection method, and gas permeation method are usually difficult to apply. With the development of computer and vision technology, image-based analyses have become one of the most promising techniques for identifying the concrete’s internal pore structure [40,41]. The digital image-processing techniques can use projected images or cross-sectional images to estimate gradation and analyze the data by using professional automatic image analyzers.

Herein, this study focuses on confirming the feasibility of accurately simulating the thermal conductivity of NAAC mixed with recycled concrete fine powders by COMSOL. Based on the real pore structure’s parameters (porosity, pore size and distribution) obtained by the stereo-image method, the model is established to avoid the effects of changing production processes and material properties on the pore structure and prediction of thermal conductivity. Firstly, the NAAC was prepared with recycled concrete fine powder as the auxiliary cementing material, and NAAC with different porosities and pore size distributions was produced by changing curing temperatures. The effect of porosity on the thermal conductivity and mechanical strength of NAAC is analyzed by experimental tests. Furthermore, the pore size distribution of NAAC with different porosity were characterized by stereology-image analyses, and the thermal conductivity prediction model was established by COMSOL steady-state analyses. The thermal conductivity results of COMSOL simulations, experiments and other classical theoretical models are compared; it is proved that the finite element model based on real pore structures obtained by the image method has good accuracy. Finally, the effects of porosity, pore size distribution and concrete matrix thermal conductivities on thermal conductivity of NAAC were studied, and the results offer a relationship between the pore structure and thermal conductivity of NAAC. This work provides a possible method for predicting the thermal conductivity of NAAC by examining pore structure reconstruction and using numerical simulations.

## 2. Materials and Methods

### 2.1. Materials

The raw materials of NAAC used in this study mainly include P.O 42.5 cement, lime, gypsum, fly ash, aluminum powder-based chemical expansion agent, foam stabilizer and recycled concrete fines. The cement was obtained from Ningxia Saima Cement Co. Ltd. (Ningxia, China), and its basic properties are shown in Table 1.

**Figure 1:**
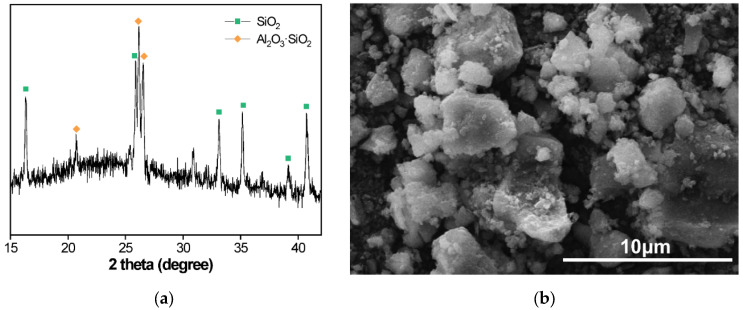
(**a**) XRD pattern and (**b**) SEM image of recycled concrete fines.

The waste concrete was screened and crushed into concrete aggregates, and then the recycled aggregates were ground by a ball mill for 50 min to obtain recycled fines. The phase composition and microstructures of the recycled concrete powder were characterized by X-ray diffraction (XRD; D/max 2550VB3, Rigaku, Japan) and scanning electron microscopes (SEM; TM4000PlusII, Hitachi, Japan), as shown in Figure 1. It can be seen that the recycled concrete fines are irregular polygons with uneven size. Grade I fly ash was used as supplementary cementitious material, and the chemical components of cement, fly ash and recycled concrete fines used in this study are presented in Table 2. According to the chemical composition, CaO and SiO_2_ are the main components of the powder material with a small number of impurities. The results show that the chemical composition of recycled concrete fines is similar to that of cement, and it can be used as a siliceous material to prepare NAAC.

The expansion agent was a GLS-65 aluminum powder-based expansion agent provided by Huaian Xinshengfa New Material Co. Ltd. (Huaian, China), and its technical index is shown in Table 3. Sodium stearate was used as a foam stabilizer, and its purity was of analytical grade. Lime and gypsum were provided by Ningxia Jinxin Environmental Technology Co. Ltd. (Ningxia, China), which complies with the Chinese standard of JC/T 621—2009.

### 2.2. Specimen Preparation and Mix Design

**Figure 2:**
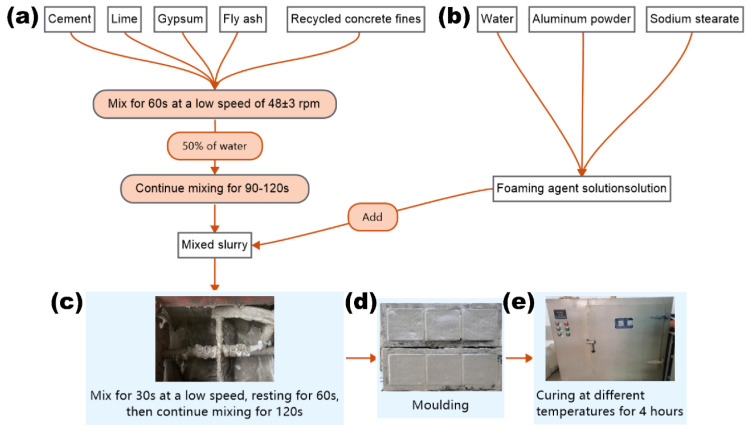
The preparation process of NAAC.

The NAAC was prepared in the following sequence (as shown in Figure 2): Cement, lime, gypsum, fly ash and recycled concrete fines were added into the mixer and mixed at low speeds (48 ± 3 rpm) for 1 min. Then, 50% of the water required for mixing was introduced into the mixture of dry ingredients and mixed for 1.5–2 min to obtain a well-mixed slurry. After that, aluminum powder and sodium stearate were mixed in the remaining mixing water, and then the prepared aqueous solution was added into the resulting mixture slurry; mixing continued for 0 s. After resting for 1 min, the slurry was continually mixed at low speeds for 2 min until a homogeneous mixture without agglomerates was obtained.

For the measurement of thermal conductivity, the slurry in a fresh state was poured into pre-prepared 100 mm × 100 mm × 100 mm molds brushed with lubricants and compacted with a metal rod. The molds with concrete were then placed into an oven with a relative humidity of 40 ± 5% for 4 h, and the curing temperatures were controlled at T1 (30 °C), T2 (35 °C), T3 (40 °C), T4 (45 °C) and T5 (50 °C), respectively. After the NAAC formed and expanded, the increased volume was removed by using scraper. Next, the specimens were covered with plastic sheeting and kept indoors at a temperature of 22 ± 2 °C for 24 h. Finally, the specimens were demolded and naturally cured to the specified age for performance testing. To increase the reliability of test results, three samples of each mixture were made.

The reference concrete was used for testing the thermal conductivity of the concrete matrix, and the mix proportions of the reference concrete and NAAC are shown in Table 4.

### 2.3. Pore Structure Characterization

#### 2.3.1. Pretreatment of Specimen

In this study, the porosity and pore size distribution of NAAC were characterized by the image-based analysis method. In order to improve the measurement accuracy, the image-based analysis of NAAC’s pore structure requires preliminary processing, including contrast enhancements. First, the specimens that have been molded and fully cured were cut, and the exposed surfaces after cutting should be parallel and flat. Then, each surface of the test block was polished with 400-, 2000- and 5000-mesh sandpaper to make the exposed surfaces smooth and flat, and the pore structure was clearly visible. Finally, the cutting surfaces were evenly smeared with ink and dried naturally. The gypsum powder was used to fill the pores on the cutting surface, and the excess gypsum powder was removed with a scraper to obtain a high contrast specimen surface.

#### 2.3.2. Stereology Principle

Stereology analysis is a mathematical method for studying the relationship between s-dimensional measurements obtained from the section of an organization and its n-dimensional (where s < n) parameters, which allows inferring the three-dimensional structural characteristics of NAAC by analyzing its two-dimensional structural features [42,43]. The fundamental viewpoint of stereology is that the structure of a material can be considered as an image consisting of points, lines, planes and bodies. There is a quantitative relationship between the statistics of a phase at point P, line length L, plane area A and volume V. The basic stereological Equation (1) applied here is as follows.

V_V_ = A_A_ = L_L_ = P_P_, (1)

The component area fraction A_A_ in a NAAC section is an unbiased estimate of the component volume fraction V_V_ in a three-dimensional structure. Consequently, the statistical results of the two-dimensional distribution can be used to characterize the three-dimensional pore distribution.

#### 2.3.3. Image Processing

**Figure 3:**
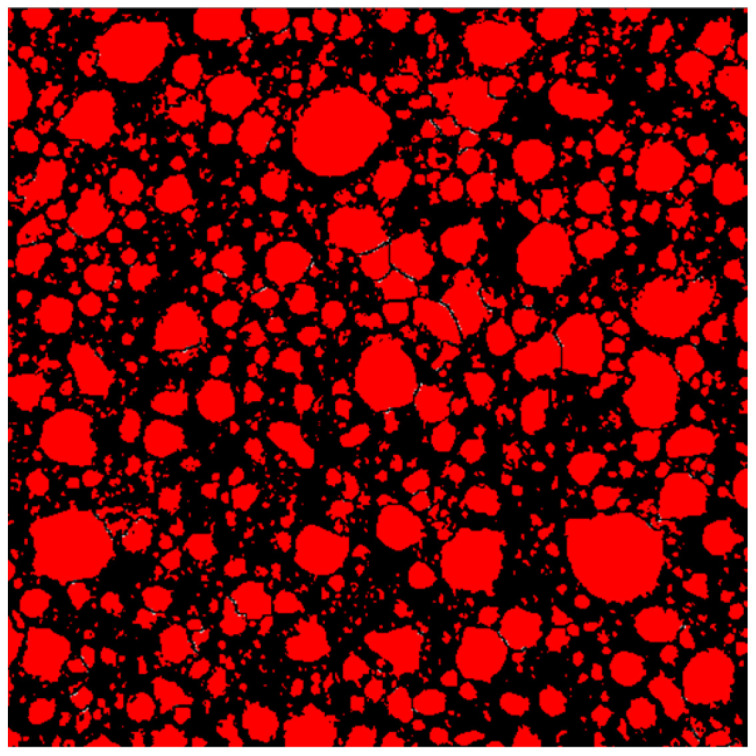
Cross section of NAAC after Image-Pro Plus software processing.

The image-processing software and pore structure characterization software used in this study was Image-Pro Plus [44]. First, the cross sections of NAAC specimens after pretreatment were photographed and cropped using a digital microscope. The maximum resolution of the obtained RGB images was 640 × 480 using 50× magnification. Then, the Image-Pro Plus software was used for image binarization and pore segmentation to obtain the image shown in Figure 3. The red part represents pore spaces, and the black part represents pore walls.

### 2.4. Thermal Conductivity Measurement

**Figure 4:**
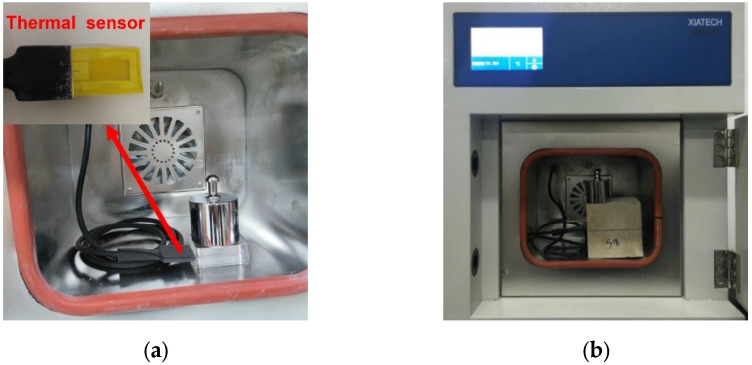
(**a**) Automatic test instrument for measuring thermal conductivity with hot wire method; (**b**) Thermal conductivity test of NAAC.

The thermal conductivity was measured by the transient hot-wire method, and the instrument used was a TC3000E heat conduction coefficient measurement device (measuring range is 0.001–50.0 W/mK, measuring accuracy is 0.0005 W/mK), as shown in Figure 4. Before formal measurements, the standard sample was used to calibrate the instrument. The specimens that have reached the specified curing age were cut into two equal volumes with flat surfaces, and the residual powder was removed. Then, the sensor was placed in the middle of the cut sample, and a 500 g weight was placed above the sample to produce the two parts after cutting them closely together so as to reduce test errors. The test voltage was 1.5 v, and each specimen was tested five times with an interval of 10 s.

### 2.5. Numerical Modeling

The COMSOL finite element software can effectively simulate the pore structure and predict the thermal conductivity of composite materials. A steady-state heat conduction-based numerical model was developed by using COMSOL to simulate the thermal conductivity of NAAC with different porosity and pore size distribution.

#### 2.5.1. Principles of Heat Conduction and Material Parameters

Heat conduction, convection and radiation are the basic methods for heat transfers. The heat transfer process relative to porous materials from the high temperature section to the low temperature section is a result of a contribution of these three different mechanisms. The NAAC is a two-phase material with a concrete matrix and air; the internal heat transfer process comprises heat conduction in a cementitious matrix, heat conduction, heat convection and heat radiation in pores. Due to the small pore size inside the NAAC, it is difficult for the air to circulate; thus, the heat transfer due to convection can be ignored. Furthermore, radiation usually accounts for a small proportion in the heat transfer of porous concrete and can be negligible. Consequently, the heat transfer simulation of NAAC in this study only considered the heat conduction. The thermal conductivity of the concrete matrix was measured by experiments, and the parameters of the concrete matrix and air are shown in Table 5.

#### 2.5.2. Calculating Model of Thermal Conductivity

**Figure 5:**
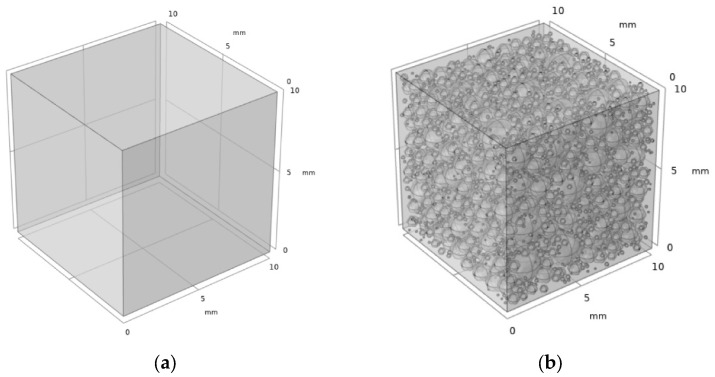
The three-dimensional models of the (**a**) reference concrete and (**b**) NAAC (curing temperature of 35 °C as an example).

As mentioned in the introduction, the existing analysis models are usually based on the random generation method for reproducing the microstructure and the pores forming randomly based on some main structural parameters. In such models, the realistic influences of production processes and material properties on pore size and distribution are usually neglected. In this paper, the three-dimensional model of the NAAC was established by COMSOL based on the porosity and pore size distribution obtained from the pore structure’s characterization. Considering the computational volume and time, the specimen size used for simulation was 10 mm × 10 mm × 10 mm, and the pores are described as spheres in the solid matrix [45]. The conditions for the formation of pore structure were that the pores were within the range of the concrete specimen, and the pores did not overlap or stick together. Due to the fineness requirement of meshing, the lower limit of the pore size was set as 0.1 mm. The three-dimensional models of the reference concrete and NAAC are shown in Figure 5.

The model was established by mimicking the actual thermal conductivity test environment, and the heat flux was set to be transferred from the upper side to the lower side of the sample without internal heat generation. The parameter settings are as follows: the room ambient temperature was set at 20 °C. The upper boundary perpendicular to the *z*-axis was set at 35 °C (hot boundary), and the lower boundary was set at 15 °C (cold boundary). Additionally, the other four boundaries parallel to the *z*-axis were set as open boundaries.

**Figure 6:**
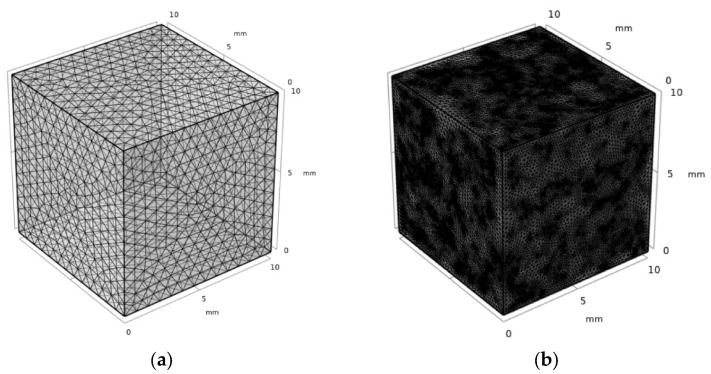
The meshing results of (**a**) reference concrete and (**b**) NAAC (curing temperature of 35 °C as an example).

The meshing method with physical field controls in COMSOL was used, and the grid’s independence test was carried out. It was determined that the simulation results of the thermal conductivity of NAAC are less sensitive to grid fineness. Based on the consideration of the simulation accuracy of small size pores and calculation time, the meshing parameters of reference concrete were set as follows: the maximum unit size of reference concrete was 1 mm, the minimum unit size was 0.18 mm, the maximum unit growth rate was 1.5, the curvature factor was 0.6, and the narrow area resolution was 0.5. The meshing parameters of NAAC were set as follows: The maximum unit size of reference concrete was 0.8 mm, the minimum unit size was 0.1 mm, the maximum unit growth rate was 1.45, the curvature factor was 0.5, and the narrow area resolution was 0.6. The meshing results of the reference concrete and NAAC are shown in Figure 6.

## 3. Results and Discussion

### 3.1. Effect of Porosity on Thermal Conductivity and Compressive Strength

**Figure 7:**
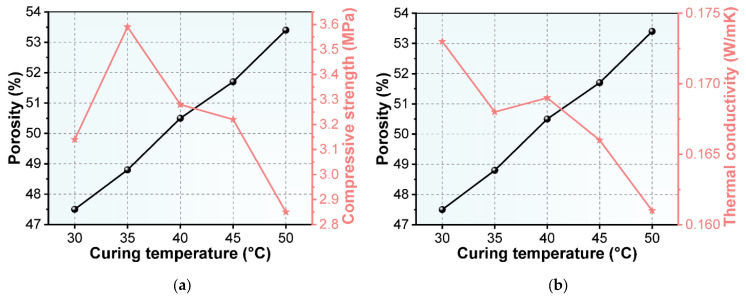
(**a**) Porosity and thermal conductivity of NAAC at different curing temperatures; (**b**) porosity and compressive strength of NAAC at different curing temperatures.

The porosity, compressive strength and thermal conductivity of the NAAC at different curing temperatures were tested, and the results are shown in Figure 7. The results show that the curing temperature of NAAC has a significant effect on its porosity. With the increase in curing temperature, the porosity of NAAC increases. As observed from Figure 7a, the compressive strength of NAAC firstly increases and then decreases with the increase in porosity, showing a downward trend as a whole. With the increase in the volume of pores, the compressive strength of NAAC decreases, which is in line with the basic law of porous materials. The compressive strength increases as the porosity increases from 47.5% (35 °C) to 48.8% (40 °C). This is mainly because the compressive strength of NAAC is not only affected by the porosity but also the pore size and pore distribution. The NAAC with a uniform distribution of pore structure, concentrated pore size distribution and a smaller number of connected pores and large pores show better compressive properties. Furthermore, the experimental thermal conductivity of the reference concrete (with approximately 0 porosity) is 0.3414 W/mK. It can be seen from Figure 7b that the thermal conductivity of NAAC is much lower than that of the reference concrete, and it decreases with the increase in porosity. This is mainly because the NAAC is a two-phase composite material of air and concrete. The thermal conductivity of air under standard conditions is only 0.023 W/mK, which is much lower than that of the concrete matrix (an order of magnitude difference). Therefore, the increase in the proportion of air in the unit volume of NAAC can reduce the overall thermal conductivity.

**Figure 8:**
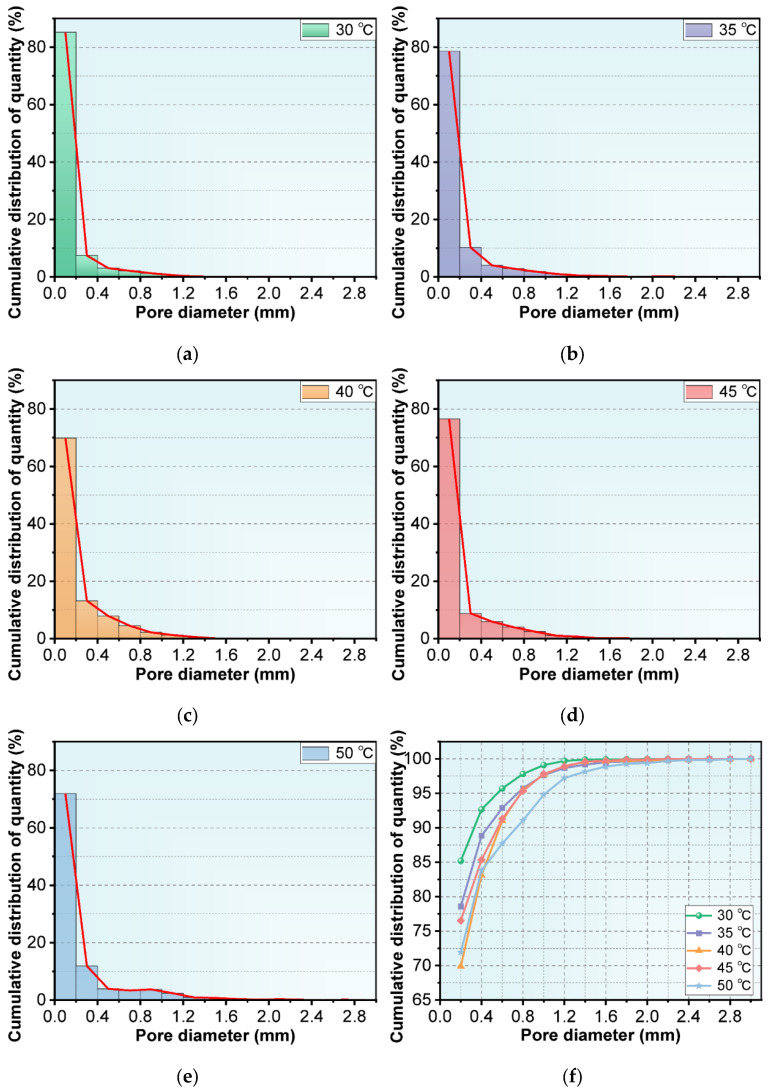
Pore number distribution at (**a**) 30 °C, (**b**) 35°C, (**c**) 40 °C, (**d**) 45 °C and (**e**) 50 °C; (**f**) cumulative frequency distribution of the pore number at different curing temperatures.

**Figure 9:**
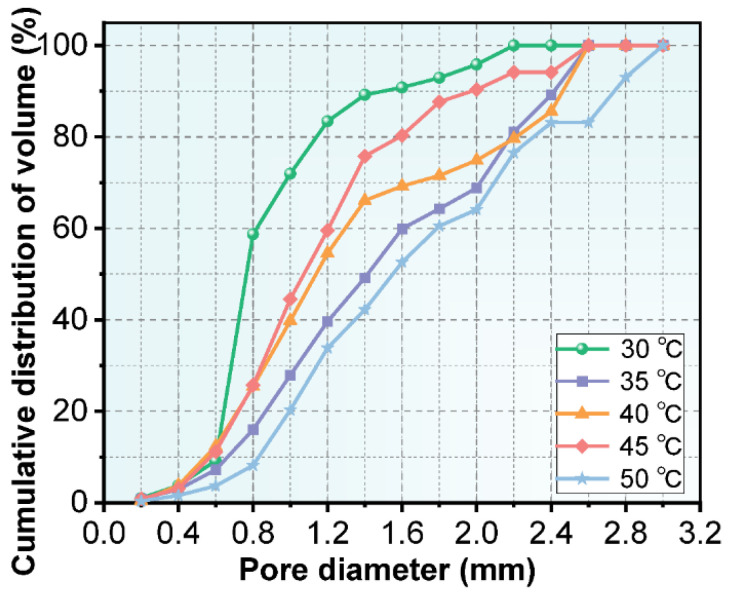
Cumulative frequency distribution of the pore volume at different curing temperatures.

The thermal conductivity of NAAC are mainly attributed to the porosity and pore size distribution. The pore size distribution of NAAC mixed with recycled fines at five different curing temperatures was statistically analyzed by Image Pro Plus software, and the cumulative frequency distribution of the number of pores at each level is shown in Figure 8. It can be seen from Figure 8a–e that the number of small pores less than 0.2 mm is significantly higher than that of other pore sizes, reaching 85.19%, 78.59%, 69.88%, 76.53% and 71.93%, respectively. From Figure 8f, it can be obtained that the D90 of NAAC at five different temperatures is 0.4 mm (92.7%), 0.6 mm (92.9%), 0.6 mm (91.0%), 0.6 mm (91.3%) and 0.8 mm (91.1%) (D90 represents the pore diameter when the cumulative pore distribution is 90%), which proves that the diameter of most internal pores is less than 0.8 mm. A distinct phenomenon worth noticing is that the pore size of NAAC tends to increase significantly with the increase in curing temperatures. Furthermore, the D100 of NAAC at five temperatures is 2.2 mm, 2.6 mm, 2.6 mm, 2.6 mm and 3.0 mm, respectively. It can be seen that with the increase in curing temperatures, the maximum pore diameter of NAAC gradually increases. The reason behind this phenomenon is that with the porosity of NAAC increases with the increase in curing temperature. As a result, the probability of contact and fusion between pores increases, and there is a higher chance to generate large pore sizes. Further statistical analyses of the cumulative frequency distribution of the pore volumes at all levels were performed, and the results are shown in Figure 9. It can be seen that the D10 of NAAC at five temperatures is 0.6 mm (9.2%), 6 mm (7.2%), 0.6 mm (12.3%), 0.6 mm (11.2%) and 0.8 mm (8.2%). Moreover, the D90 is 1.6 mm (90.8%), 2.4 mm (89.2%), 2.4 mm (85.6%), 2.0 mm (90.4%) and 2.8 mm (93.1%), respectively. This comparison indicates that with the increase in pouring temperatures, the volume proportion of large pore sizes also increases. Based on the above analysis, with the increase in curing temperatures, the porosity increases and the pore size tends to be larger. Therefore, the thermal conductivity decreases, which is consistent with the above thermal conductivity test results.

### 3.2. Simulation of Thermal Conductivity

#### 3.2.1. Steady-State Thermal Analyses

**Figure 10:**
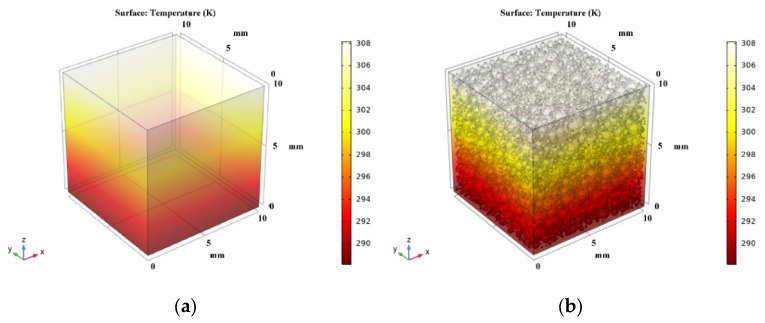

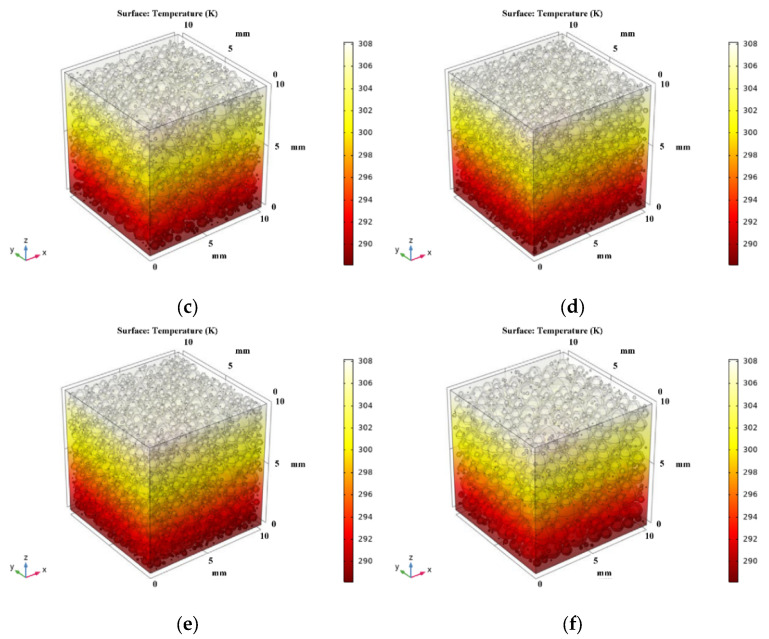
(**a**) Temperature distribution of reference concrete; temperature distribution of NAAC specimens at (**b**) 30 °C, (**c**) 35°C, (**d**) 40 °C, (**e**) 45 °C and (**f**) 50 °C.

**Figure 11:**
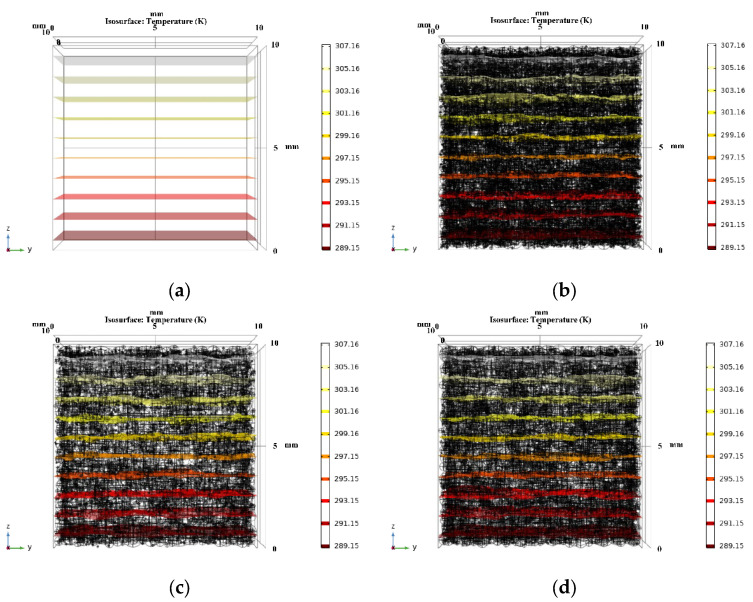

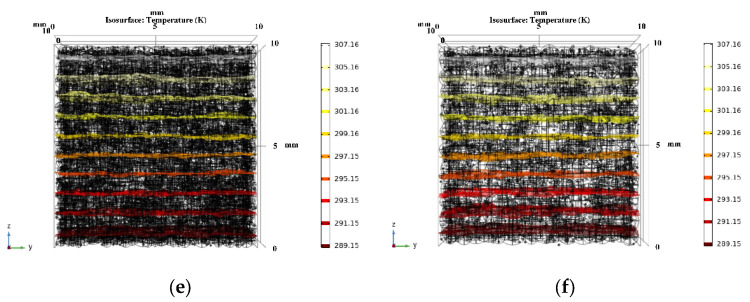
(**a**) Isotherms of reference concrete; isotherms of NAAC specimens at (**b**) 30 °C, (**c**) 35 °C, (**d**) 40 °C, (**e**) 45 °C and (**f**) 50 °C.

Based on the porosity and pore size distribution obtained from the pore structure’s analysis, the model of the NAAC’s pore structure was reconstructed by COMSOL. Moreover, the three-dimensional temperature distribution of NAAC specimens at different curing temperatures was obtained using COMSOL steady-state simulations, as shown in Figure 10. It can be seen that the temperature distribution of NAAC specimens obtained under different curing temperatures is similar to that of the reference concrete, and the NAAC with different porosities all reach the temperature equilibrium in the middle of the test blocks. Figure 11 shows the three-dimensional isotherm of the NAAC test blocks. It can be seen from Figure 11a that the isotherm of the reference concrete specimen (porosity is approximately 0) is straight and uniformly distributed, which indicates that heat is evenly diffused in the concrete test block. It is worth noting that with the increase in curing temperatures (porosity increases), the isotherms between the continuous medium and the dispersed medium are significantly distorted (as shown in Figure 11b–f). This is mainly due to the increase in porosity and the appearance of large pore sizes as the curing temperature increases. The change in the pore’s structure will lead to an inhomogeneous interface and changes in the heat transfer path. Additionally, the thermal conductivity of air is much lower than that of concrete, and there will be a thermal bridge effect at the edges of the pore’s structure.

#### 3.2.2. Calculation of Thermal Conductivity

According to the results of the steady-state thermal analysis, the temperature gradient and heat flux of NAAC with different porosities can be obtained by the steady-state heat conduction equation, which is expressed as Equation (2). According to Fourier’s law, the thermal conductivity of NAAC can be further calculated, and the calculation method is shown in Equation (3):

(2) $$\rho C_{p}u\cdot \nabla T+\nabla \left(-k\nabla T\right)=Q,$$

(3) q=−k∇T,

where *u* is the velocity field (m/s), ρ is the material density (kg/m^3^), *C_p_* is the specific heat capacity of material (Jkg^−1^K^−1^), ∇ is the gradient operator, *T* is the temperature (K), *Q* is the quantity of heat (J), *k* is the thermal conductivity (W/mK), and *q* is the heat flux (W/m^2^).

**Figure 12:**
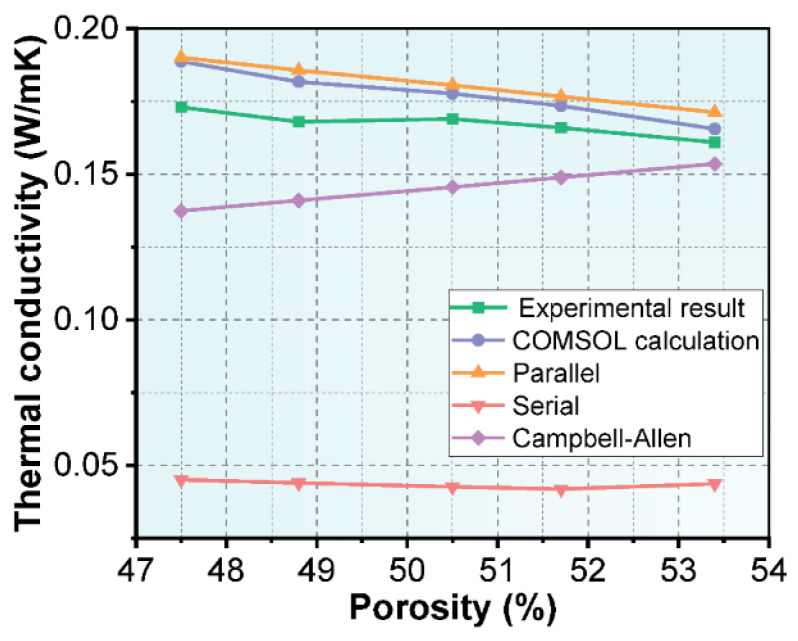
Comparison between COMSOL analysis, classical thermal conductivity models and test results.

The series model, the parallel model and the Campbell-Allen model derived from Ohm’s law based on the series and parallel model are the classic models for thermal conductivity calculations. The thermal conductivity of NAAC with different porosities calculated by COMSOL was compared with the predicted results of the classical models, and the results are shown in Figure 12. Table 6 further shows the relative errors between the predicted thermal conductivity of the four models and the experimental test results. It can be seen that the thermal conductivity predicted by COMSOL is in the best agreement with the experimental test results, and the errors under the five types of porosity are 9.09%, 8.18%, 5.17%, 4.53% and 2.84%. A distinct phenomenon worth noticing is that the prediction accuracy of COMSOL increases with the increase in NAAC’s porosity. This result indicates that the COMSOL finite element software has a better adaptability to the dense and complex pore structure of NAAC. The high accuracy of COMSOL in predicting the thermal conductivity of NAAC can be attributed to the fact that the model was established based on the information on porosity and pore size distribution obtained by the image method, and the basic parameters such as material density and specific heat capacity were fully considered [46,47]. Simultaneously, the COMSOL finite element software can well describe the distribution of the internal pore structure of NAAC and effectively mesh so as to obtain the quantitative results of the thermal conductivity. In addition to the COMSOL simulation, the parallel model also shows good predictions. The reason is that the porosity of NAAC is large and the pore size distribution is relatively concentrated, so its internal pore structure is closer to the geometric model of the parallel model than that of the series model and the Campbell-Allen model. The above results show that COMSOL simulations can be used as effective methods for predicting the thermal conductivity of NAAC, and its calculation accuracy is higher than that of simple two-phase models, such as parallel models, series models and the Campbell-Allen model. Moreover, the COMSOL simulation shows better prediction results on the thermal conductivity of NAAC with high porosity.

### 3.3. Study on the Factors Affecting the Thermal Conductivity

#### 3.3.1. Effect of Pore Size Distribution on Thermal Conductivity

The pore size distribution is also an important parameter that affects the internal pore structure of NAAC. Thus, the influence of pore size distribution on the thermal conductivity of NAAC was investigated by controlling the change in pore size in an interval. First, the pore size was controlled in a small interval of 0.2 mm, and the effect of the pore size distribution interval on the thermal conductivity was investigated by changing the pore size distribution’s center. The results of the COMSOL simulation are shown in Table 7, and it can be seen that the thermal conductivity of NAAC varies very slightly with the change in distribution center. Furthermore, the 1.5 mm was used as the center of the pore size distribution interval, and the size of the interval gradually changed to control the dispersion coefficient of the pore size. The prediction results of the thermal conductivity are shown in Table 8, which proves that the dispersion degree of the pore size in the pore size’s distribution area centered at 1.5 mm has little effects on the thermal conductivity of NAAC. According to the above analysis, when the porosity of NAAC is determined, the thermal conductivity is less sensitive to the pore size’s distribution. This conclusion is consistent with the results of relevant studies; that is, the influence of pore diameter on thermal conductivity can be ignored.

#### 3.3.2. Effect of Concrete Matrix Thermal Conductivity on NAAC Thermal Conductivity

The thermal conductivity of the concrete matrix is usually measured by laboratory tests, and the results are usually subject to large errors. Therefore, it is necessary to consider the influence of the concrete matrix’s thermal conductivity on the prediction accuracy of NAAC’s thermal conductivity. The thermal conductivity of the concrete matrix was changed so that it can deviate from the experimental measurement results (with an error range from −15 to 15%), and the sensitivity of the thermal conductivity of NAAC to its variation was explored. Simulations were performed with parameters obtained for NAAC at T2 temperatures, and the simulation results are shown in Table 9.

**Figure 13:**
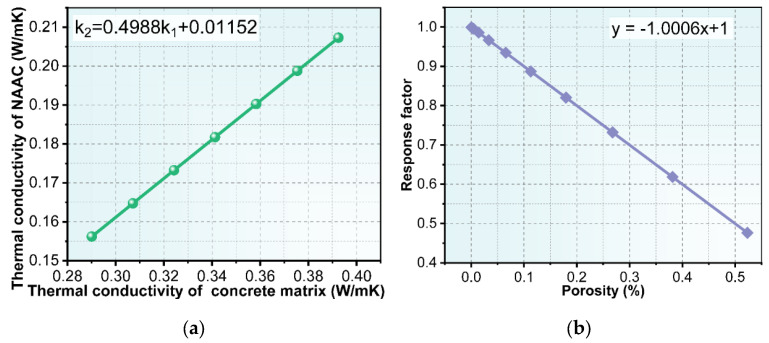
(**a**) Effect of concrete matrix thermal conductivity on the thermal conductivity of NAAC; (**b**) response coefficient of the NAAC thermal conductivity with different porosities to the concrete matrix’s thermal conductivity.

Figure 13a shows the influence of the concrete matrix’s thermal conductivity on the thermal conductivity of NAAC, and it can be seen that the two are basically linear. The fitting result is k_2_ = 0.4988k_1_ + 0.01152, and it has an error coefficient, SSE, of 5.718 × 10^−11^ and R^2^ of 1, which indicates that there is a strong linear correlation between the two. The slope of k_1_ to k_2_ was used as the response coefficient of the NAAC thermal conductivity to the concrete matrix’s thermal conductivity, as shown in Figure 13b. It can be seen that the porosity of NAAC is linearly and negatively correlated with the degree of responses, and the rate of change is close to 1. Consequently, with the increase in porosity, the sensitivity of NAAC thermal conductivity to the concrete matrix’s thermal conductivity decreases.

## 4. Conclusions

In this study, recycled concrete fine powder was used to prepare NAAC. The NAAC with different porosities and distributions was fabricated by the non-autoclaved method at different curing temperatures, and its pore size distribution was reproduced by stereology-image analysis. Based on these pore structural parameters, the thermal conductivity prediction model was established by the COMSOL steady-state heat transfer module. The established model was used to study the effects of porosity pore size distributions and concrete matrix thermal conductivity on the thermal conductivity of NAAC. The following conclusions can be drawn:

(1) Recycled concrete fine powder can be used as a good auxiliary cementitious material to produce NAAC, and the curing temperature of the non-autoclaved method has a great effect on the pore structure of NAAC. With the increase in curing temperature, the porosity of NAAC increases and the number and volume proportion of macropores increase. The increase in porosity will lead to a decrease in thermal conductivity and the compressive strength of NAAC.
(2) The thermal conductivity results of COMSOL simulations, experiments and other classical theoretical models were compared, and it was found that the error between the COMSOL simulations and the experiments was less than 10% under different porosities, which is smaller than other models and has strong reliability. The prediction accuracy of COMSOL increases with the increase in NAAC porosity. This shows that COMSOL simulations can provide a fine description of the distribution of the complex pore structure of NAAC. Compared with the model that only considers porosity and adopting random pore distributions, the COMSOL model based on the real porosity and pore size distribution obtained by the image method can fully consider the impact of production processes and material properties on the pore structure of NAAC, and the accuracy of thermal conductivity estimates is improved.
(3) The steady thermal conductivity of NAAC is less sensitive to the distribution and dispersion of pore size in a given porosity. With the increase in porosity, the thermal conductivity of NAAC is linearly and negatively correlated with that of the concrete matrix, and the correlation is close to 1.
(4) This study provides a possible method for predicting the thermal conductivity of NAAC from the point of view of pore structure reconstruction and numerical simulations, and the established model has good accuracy. Further research can explore the use of machine learning and 3D reconstruction technology to reconstruct the three-dimensional model of a pore structure so as to establish a faster and more accurate calculation method for thermal conductivities.

## Figures and Tables

**Table 1:** Basic properties of cement.

Cement Grade	Specific Surface Area (m^2^/kg)	Setting Time (min)		Water Consumption of Normal Consistency (%)	LOI (%)
		Initial	Final		
P.O 42.5	350	182	234	27.2	4.7

**Table 2:** Chemical components of raw materials.

Constituents (wt.%)	CaO	SiO_2_	Al_2_O_3_	Fe_2_O_3_	SO_3_	Na_2_O	LOI
Cement	43.5	14.1	5.19	2.17	4.91	21.3	8.83
Fly ash	5.7	51.5	24.8	7.0	1.5	1.7	7.8
Recycled concrete fines	34.1	23.9	3.86	2.32	1.32	27.7	6.8

**Table 3:** Characteristics of aluminum powder-based expansion agent.

Label	Type	Solid Content (%)	Active Aluminum in Solid (%)	Rate of Gas Generation (%)		
				4 min	16 min	30 min
GLS-65	Water-based	65	85	40–60	≥90	≥99

**Table 4:** Mix proportions of each sample.

Mix Types	W/C	Cement (%)	RCFP (%)	Gypsum (%)	FA (%)	Lime (%)	Expansion Agent (%)	Foam Stabilizer (%)
Reference Concrete	0.65	20	14	18	46	2	-	-
NAAC	0.65	20	14	18	46	2	0.06	0.12

Note: W/C represents the water-cement ratio; RCFP represents the recycled concrete fine powder; FA represents the fly ash.

**Table 5:** Phase parameters of NAAC.

Phase	Thermal Conductivity (W/mK)	Specific Heat Capacity (J/(kg∙K))	Density (kg/m^3^)
Concrete matrix	0.3414	1050	2470
Air	0.023	1000	1.29

**Table 6:** Relative errors between the thermal conductivity of the COMSOL analysis, classical thermal conductivity models and test results.

Curing Temperature (°C)	Porosity (%)	Experimental (W/mK)	COMSOL (W/mK)	Parallel (W/mK)	Serial (W/mK)	Campbell-Allen (W/mK)
30 °C	47.5	0.173	0.18873 (9.09%)	0.1901 (9.88%)	0.0451 (−73.93%)	0.1374 (−20.58%)
35 °C	48.8	0.168	0.18175 (8.18%)	0.1857 (10.54%)	0.0440 (−73.81%)	0.1410 (−16.07%)
40 °C	50.5	0.169	0.17773 (5.17%)	0.1806 (6.86%)	0.0427 (−74.73%)	0.1456 (−13.85%)
45 °C	51.7	0.166	0.17352 (4.53%)	0.1767 (6.45%)	0.0419 (−74.76%)	0.1489 (−10.30%)
50 °C	53.4	0.161	0.16558 (2.84%)	0.1713 (6.40%)	0.0437 (−72.86%)	0.1536 (−4.60%)

**Table 7:** Effect of pore diameter distribution on thermal conductivity.

Distribution interval (mm)	0.4–0.6	0.6–0.8	0.8–1.0	1.0–1.2	1.2–1.4	1.4–1.6
Interval center (mm)	0.5	0.7	0.9	1.1	1.3	1.5
Thermal conductivity (W/mK)	0.26241	0.26281	0.26277	0.26263	0.26252	0.26270
Distribution interval (mm)	1.6–1.8	1.8–2.0	2.0–2.2	2.2–2.4	2.4–2.6	2.6–2.8
Interval center (mm)	1.7	1.9	2.1	2.3	2.5	2.7
Thermal conductivity (W/mK)	0.26257	0.26253	0.26204	0.26221	0.26236	0.2624

**Table 8:** Effect of pore diameter dispersion coefficient on thermal conductivity.

Distribution interval (mm)	0.2–2.8	0.4–2.6	0.6–2.4	0.8–2.2	1.0–2.0	1.2–1.8	1.4–1.6
Coefficient of dispersion	0.61710	0.45339	0.33806	0.27670	0.20859	0.13818	0.09101
Thermal conductivity (W/mK)	0.26283	0.26281	0.26277	0.26273	0.26278	0.26256	0.26266

**Table 9:** Effect of concrete matrix thermal conductivity on the thermal conductivity of NAAC.

Error	−15%	−10%	−5%	0%	5%	10%	15%
Thermal conductivity of concrete matrix (k_1_)	0.29011	0.30717	0.32424	0.3413	0.35837	0.37543	0.39250
Thermal conductivity of NAAC (k_2_)	0.15622	0.16473	0.17325	0.18175	0.19027	0.19878	0.20729

## Data Availability

The data used to support the findings of this study are available from the corresponding author upon request.
